# Time to resolution of tubal ectopic pregnancy following methotrexate treatment: A retrospective cohort study

**DOI:** 10.1371/journal.pone.0268741

**Published:** 2022-05-24

**Authors:** Marcus J. Davenport, Anthea Lindquist, Fiona Brownfoot, Natasha Pritchard, Stephen Tong, Roxanne Hastie

**Affiliations:** 1 Department of Obstetrics and Gynaecology, Mercy Hospital for Women, Heidelberg, Victoria, Australia; 2 Mercy Perinatal, Mercy Hospital for Women, Heidelberg, Victoria, Australia; 3 Translational Obstetrics Group, Department of Obstetrics and Gynaecology, University of Melbourne, Heidelberg, Victoria, Australia; Universita degli Studi dell’Insubria, ITALY

## Abstract

**Objective:**

To determine the time to resolution of tubal ectopic pregnancy after methotrexate treatment.

**Methods:**

A 14-year retrospective cohort study was performed from 2004–2018 and assessed 216 women treated with single-dose methotrexate for tubal ectopic pregnancy. Women were treated using a single-dose protocol of intramuscular methotrexate (50mg/m^2^) for confirmed tubal ectopic pregnancy on ultrasound. Ectopic pregnancies were included if the ectopic pregnancy mass was <35mm, no evidence of rupture and no embryonic cardiac activity. Serum hCG was measured on day 1, 4 and 7 of treatment and then at standard weekly intervals until resolution. Where there was not a ≥15% decline in hCG from day 4 and day 7, a second dose of methotrexate was administered. The primary outcome was time to resolution (days), with serum hCG <5 IU/L considered resolved. The secondary outcome was need for rescue surgery.

**Results:**

Among women who did not proceed to surgery, the median time to resolution was 22 days (IQR 14,34). Time to resolution and need for rescue surgery increased with baseline hCG. When hCG was <1000 IU/L, the median was 20 days (IQR 13,29) but 34.5 days (IQR 22,48) with hCG >2000 IU/L. Early hCG trends were predictive of time to resolution and likelihood of rescue surgery; a hCG rise of >1000 IU/L between Days 1–4 increased time to resolution to 61 days (IQR 35,80) and an odds ratio of rescue surgery of 28.6 (95% C.I. 5.3,155.4).

**Conclusion:**

The median time to resolution for ectopic pregnancies treated with methotrexate is 22 days and associated with baseline hCG levels. The predictive value of baseline hCG may be useful in clinical decision making and counselling women considering methotrexate for ectopic pregnancy.

## Introduction

Ectopic pregnancy affects 1–2% of pregnancies [1] and is the leading cause of maternal death in the first trimester, responsible for 6% of all maternal mortality [2]. Over 98% of ectopic pregnancies occur within the Fallopian tubes [3], however they do also occur on the ovary, the cornua or interstium of the uterus and even intra-abdominally [4].

Methotrexate is a folic acid antagonist first introduced in the management of ectopic pregnancy in the early 1980s [5]. In select patients, methotrexate may be preferred over surgery (laparoscopic salpingectomy) as it spares the Fallopian tube and subsequently avoids the small but potentially serious risks of surgery [6]. Today, single-dose methotrexate at 50mg/m^2^, is well-established as a treatment for unruptured ectopic pregnancy and serum human chorionic gonadotrophin (hCG) levels are tracked until the ectopic pregnancy is resolved [6, 7]. Although ultrasound is useful in diagnosing tubal ectopic pregnancies, they have limited utility in predicting treatment success and resolution [8, 9].

When weighing up whether to proceed to definitive surgical excision or to opt for medical management, women often wish to know how long it will take for the ectopic pregnancy to resolve. Until the ectopic has fully resolved, regular clinic visits and close monitoring of symptoms that may indicate the ectopic has ruptured are required [7]. Furthermore, such information may also be valuable in guiding clinical decision making.

Although time resolution following medical management of ectopic pregnancy would be useful clinically, currently, it is poorly characterized. The American College of Obstetricians and Gynaecologists report that resolution following methotrexate is usually 2–4 weeks but may take up to 8 weeks [7].

Baseline (pre-treatment) hCG concentrations correlate with treatment success [10]. Early hCG trends between days 1 to 4 after the initial methotrexate treatment are also associated with success [11–13]. We therefore hypothesize that for medical management with methotrexate, the impact of pre-treatment hCG values and it’s trends may also predicate time to resolution.

This study aims to investigate time to resolution for tubal ectopic pregnancies following medical management via methotrexate treatment and the effect of pre-treatment hCG values and trends from day 1 to 4 of treatment on resolution times.

## Methods

This retrospective cohort study included 216 women initially treated with single-dose methotrexate for a confirmed tubal ectopic pregnancy at a tertiary obstetrics and gynaecology centre in Melbourne, Australia between 2004 to 2018. Ethical approval for this study was obtained from Mercy Health Human Research Ethics Committee on September 13, 2018. The institutional review board waived the need for obtaining informed consent from individual patients.

### Inclusion and exclusion criteria

Women were eligible for inclusion if they had a confirmed tubal ectopic pregnancy on ultrasound and received single-dose methotrexate (at 50mg/m^2^) as per previous protocols [14]. Women were excluded with a pregnancy of unknown location, that is without a confirmed ultrasound diagnosis of an ectopic pregnancy but is suspected based on plateauing hCG levels.

At our institution, women were eligible for single-dose methotrexate if the ectopic pregnancy mass was <35mm, with no evidence of tubal rupture, no embryonic cardiac activity or co-existing intrauterine gestation and if their baseline hCG was <3500 IU/L.

Serum hCG was measured on the day of treatment (day 1) and repeated on day 4 and 7. If there was a ≥15% decline in hCG from day 4 and day 7, titres were repeated weekly until the ectopic pregnancy resolved [15]. If there was a <15% decline in hCG, patients were eligible for a second dose methotrexate. If there was a ≥15% decline in hCG from day 4 to day 7 after the second dose of methotrexate, titres were repeated weekly until resolution.

Rescue surgery was indicated if women failed methotrexate therapy or there was evidence of tubal rupture. Rupture was considered in women presenting with severe pain, haemodynamic instability, a significant drop in haemoglobin or sonographic evidence of hemoperitoneum. Women with poor response to second dose methotrexate, such as those with plateauing or rising hCG, were recommended surgery, as well as in women who declined a second dose of methotrexate following an insufficient decline in serum hCG from day 4 to 7.

### Outcomes

The primary outcome was time to resolution (in days) from the day of initial treatment with single-dose methotrexate. Resolution was considered when serum hCG levels were <5 IU/L.

The secondary outcome measured was need for rescue surgery. Both primary and secondary outcomes are presented in relation to pre-treatment hCG concentration and changes in hCG concentration from day 1 to 4.

### Data collection

Eligible patients were identified using a unique hospital code for patients diagnosed with tubal ectopic pregnancies. Patient medical records, including pathology and ultrasound reports, were manually audited and data collected using a standardized electronic data collection form.

### Statistical analysis

Categorical data was presented as a proportion (%) and continuous data as medians with interquartile range (IQR). ANOVA was used for comparisons across multiple groups. hCG data was log transformed and logistic regression used to determine associations between pre-treatment hCG or change in hCG and surgical excision. Data was presented as odds ratios with 95% confidence intervals. Statistical analysis was performed using STATA SE.

## Results

216 women were included in this study. As demonstrated in Table 1, women treated with methotrexate had a mean age of 32.1 years (± 4.8) and body mass index of 25.4 kg/m^2^ (±5.2). The median pre-treatment hCG was 758.8 IU/L and EP mass 18.4mm (±7.9). 20.4% of women required a second dose of methotrexate and 24.5% required rescue surgery.

**Table 1:** Patient baseline demographics and ectopic pregnancy characteristics.

Characteristics	Outcome (n = 216)
	N (%)
Age (years)	32.1 ± 4.8
Body Mass Index (BMI) (kg/m^2^) ^a^	25.4 ± 5.2
Parity	
0	104 (48.1)
1	74 (34.3)
≥2	38 (17.6)
Previous ectopic pregnancy	29 (13.4)
Ectopic pregnancy mass (mm)	18.4 ± 7.9
Pre-treatment hCG (IU/l), Median (min–max)	758.8 (148.6–4,951)
Required second dose of methotrexate	44 (20.4)
Required rescue surgery	53 (24.5)

^a^ BMI data not available for 4 patients.

Among the 163 women who did not require rescue surgery (n = 29 received a second dose of methotrexate), the median time to resolution was 22 days (interquartile range [IQR] 14, 34) and baseline hCG was positively associated with time to resolution (Table 2). Women with hCG <1000 IU/L had the shortest time to resolution at 20.0 days (IQR 13, 29), which increased to 31.5 days (IQR 21, 41) for hCG 1000–1999 and 34.5 days (IQR 22, 48) for 2000–2999 IU/L. Results were similar for women successfully treated with single-dose methotrexate, with a median time to resolution of 21 days (IQR 6, 97, n = 134) and a positive association between baseline hCG and time to resolution. A baseline hCG <1000 IU/L resulted in a median time to resolution of 17 days (IQR 6, 63), which increased to 28 days (IQR 6, 49) for hCG 1000–1999 IU/L, 34 days (IQR 14, 74) for 2000–2999 IU/L and 33.5 days (IQR 10, 97) for ≥3000 IU/L.

**Table 2:** Time to resolution and likelihood of surgical excision by pre-treatment hCG.

	Time to resolution		Rescue surgery	
	N = 163		N = 53	
Baseline hCG (IU/L)	n (%)	Days, median (IQR)	n (%)	Odds ratio (95% confidence interval)
<1000 (n = 130)	107 (65.6)	20.0 (13, 29)	23 (43.4)	1 (reference)
≥1000–1999 (n = 44)	36 (22.1)	31.5 (21, 41) ^b^	8 (15.1)	1.03 (0.43, 2.51)
≥2000–2999 (n = 25)	14 (8.6)	34.5 (22, 48) ^b^	11 (20.8)	3.66 (1.47, 9.07)
≥3000 (n = 17)	6 (3.7)	33.5 (20, 39)	11 (20.8)	8.53 (2.86, 25.42)

Time to resolution was calculated excluding 53 cases that required surgery. Odds ratios and 95% confidence intervals were calculated using logistic regression. 216 included in analysis.

^b^ denotes *P*<0.001 compared to pre-treatment hCG <1000.

The likelihood of rescue surgery also increased with pre-treatment hCG. Compared to women with a pre-treatment hCG <1000 IU/L, those with levels between 2000–2999 IU/L were almost 4-fold more likely to require surgery (odd ratio [OR] 3.66; 95% confidence interval [95%. CI] 1.47–9.07; 17.7% vs 40.4%). This increased to over 8-fold for those with hCG levels ≥3000 IU/L (OR 8.53; 95% CI 2.86, - 25.42; 17.7% vs 64.7%) (Table 2).

The early trends in hCG concentrations after the methotrexate treatment were associated with time to resolution. Women who had a decline in hCG concentrations between days 1–4, had a median time to resolution of 21 days (IQR 13, 31; n = 53). This increased to 32.5 days (IQR 26, 39; n = 58) when hCG rose between 1–999 IU/L and to 61 days (IQR 35, 80; n = 10) when the rise was ≥1000 IU/L (Table 3). Additionally, a rise in hCG by day 4 was strongly associated with rescue surgery. Compared to women with a decline in hCG, those who experienced a rise of up to 999 IU/L were over 7-fold more likely to require surgery (OR 7.50 95% CI 2.4, 23.6; 7.6% vs 37.9%), which increased to over 28-fold (OR 28.6 95% CI 5.3–155.4; 7.6% vs 70.0%) if hCG rose by over 1000 IU/L (Table 3).

**Table 3:** Time to resolution and likelihood of surgical excision by the change in hCG from Days 1 to 4.

	Time to resolution		Rescue surgery	
Change in hCG Days 1–4	n (%)	Days, median (IQR)	n (%)	Odds ratio (95% confidence interval)
Decrease	49 (55.7)	21.0 (13, 31)	4 (7.6)	1 (reference)
Increase≥0–999	36 (40.9)	32.5 (26, 39) ^c^	22 (37.9)	7.5 (2.4, 23.6)
Increase≥1000	3 (3.4)	61.0 (35, 80) ^c^	7 (70)	28.6 (5.3, 155.4)

Time to resolution was calculated excluding 53 cases that required surgery. Cases with missing day 1 or 4 hCG levels were excluded. Odds ratios and 95% confidence intervals (95% ^c^ denotes values calculated via logistic regression; 121 included in analysis.

## Discussion

Our results provide useful information about the expected time needed for resolution according to pre-treatment hCG concentrations, which has been previously poorly defined. The American College of Obstetrics and Gynaecologists provide a broad time to resolution ranging from 2–4 weeks but up to 8 weeks [7]. Time to resolution was longer when the baseline hCG concentrations was above 2000 IU/L and early trends in hCG were predictive of time to resolution and treatment success.

We found that baseline hCG levels were associated with time to resolution, with an increase from 20 days when baseline hCG levels were <1000 IU/L to 34.5 days for levels 2000–2999 IU/L. Our findings are in agreement with several other reported studies, which identified resolution between 23–31 days. However previous studies are limited in their sample size, often report both single and multi-dose treatment protocols, and do not clearly stratify time to resolution based on pre-treatment hCG concentrations [15–19].

Baseline hCG levels were also positively associated with the risk of requiring rescue surgery, particularly when baseline hCG levels were ≥3000IU/L (OR 8.53 (CI 2.86, 25.42)). Although previous studies have shown that higher initial hCG levels correspond to increased rates of surgical intervention, the magnitude of this risk is not well characterized for current treatment thresholds [10, 19, 20].

A large rise in hCG by day 4 of up to 999 IU/L was associated with a significant increase in the time to resolution (21 vs 61 days) and the likelihood of requiring rescue surgery (odds ratio 28.6).

We found trends from days 1 to 4 after methotrexate administration were able to predict time to resolution: a decline in hCG by day 4 was associated with a median time to resolution of 21 days, which increased to 32.5 days when hCG rose by up to 999 IU/L and 61 days for increases of ≥1000 IU/L. Although hCG concentrations are highly variable in the first week after methotrexate therapy [21–23], we found that in line with previous findings the change in hCG from Day 1 to Day 4 was associated with the likelihood of surgical management [11, 12, 24–28].

To the authors knowledge, ours is the largest dedicated study investigating time to resolution of ectopic pregnancies treated with single-dose methotrexate. Time to resolution is presented for clear hCG intervals at current treatment thresholds in order to improve translatability of these results into clinical practice. This is also the first study correlating trends in hCG concentrations from day 1 to 4 and overall time to resolution.

There are some limitations within our study. Firstly, the inherent retrospective nature of this study, which warrants future prospective studies to validate these findings. Additionally, although we performed a 14-year review of all tubal ectopic pregnancies at a tertiary referral centre, only 216 women were eligible for review and of these, the median baseline hCG was relatively low. This likely reflects the high prevalence of surgery for ectopic pregnancy at our centre, which may be overcome by the availability of further evidence of treatment success and time to resolution following medical management. Our findings are only applicable to those women with tubal ectopic pregnancy with baseline hCG <3500 IU/L. We also acknowledge that it is routine practice in some centres to manage women expectantly who have hCG levels <1500 IU/L [29].

This study also reports 24.5% of patients required surgical excision in this study. Existing literature demonstrates surgical intervention is required in 5–15% of cases [14, 17, 20, 30–34]. The decision to proceed to rescue surgery was at clinician discretion rather than strict clinical criteria. The higher surgical rates in our study may reflect a preference for surgical management in our institution and under-utility of second-dose methotrexate [35] and should be taken into consideration when extrapolating these findings into broader clinical practice.

Our findings provide added value and emphasis to pre-treatment hCG values for counselling women who are considering medical management. Although baseline hCG has previously been shown to correlate with treatment success, we now provide a well delineated time to resolution and required follow up. At current treatment thresholds, baseline hCG levels predict likelihood of success, both in terms of time to resolution and the risk of requiring rescue surgery.

Additionally, we were able to confirm previous reports of an increased likelihood of treatment failure with rising hCG between days 1 and 4. These findings are also useful in assisting clinical decision making and patient counselling, even while the ectopic pregnancy is being treated. For instance, if the clinician is at equipoise regarding whether to proceed with rescue surgery, an awareness of this information may be useful.

## Conclusion

The median time to resolution for ectopic pregnancies treated with methotrexate was 22 days, with the majority resolved within 5 weeks. Baseline hCG and early trends are predictive of time to resolution, with a rise above 1000 IU/L by day 4 associated with the longest time to resolution. These findings are of clinical importance and may be useful in guiding treatment decisions and setting patient expectations for likely resolution times and duration of required follow up.
